# Needs-Assessment for an Artificial Intelligence-Based Chatbot for Pharmacists in HIV Care: Results from a Knowledge–Attitudes–Practices Survey

**DOI:** 10.3390/healthcare12161661

**Published:** 2024-08-20

**Authors:** Moustafa Laymouna, Yuanchao Ma, David Lessard, Kim Engler, Rachel Therrien, Tibor Schuster, Serge Vicente, Sofiane Achiche, Maria Nait El Haj, Benoît Lemire, Abdalwahab Kawaiah, Bertrand Lebouché

**Affiliations:** 1 Department of Family Medicine, Faculty of Medicine and Health Sciences, McGill University, Montreal, QC H3S 1Z1, Canada; moustafa.laymouna@mail.mcgill.ca (M.L.);; 2 Centre for Outcomes Research and Evaluation, Research Institute of the McGill University Health Centre, Montreal, QC H4A 3S5, Canada; 3 Infectious Diseases and Immunity in Global Health Program, Research Institute of McGill University Health Centre, Montreal, QC H4A 3S5, Canada; 4 Chronic Viral Illness Service, Division of Infectious Diseases, Department of Medicine, McGill University Health Centre, Montreal, QC H4A 3J1, Canada; 5 Department of Biomedical Engineering, Polytechnique Montréal, Montreal, QC H3T 1J4, Canada; 6 Department of Pharmacy and Chronic Viral Illness Service, Research Centre of the University of Montreal Hospital Centre, Montreal, QC H2X 0A9, Canada; 7 Department of Mathematics and Statistics, University of Montreal, Montreal, QC H3T 1J4, Canada; 8 Faculty of Pharmacy, University of Montreal, Montreal, QC H3C 3J7, Canada

**Keywords:** pharmacists, HIV, chatbot, artificial intelligence, needs assessment, surveys and questionnaires, health information technology, healthcare providers, health knowledge–attitudes–practices, patient care

## Abstract

Background: Pharmacists need up-to-date knowledge and decision-making support in HIV care. We aim to develop MARVIN-Pharma, an adapted artificial intelligence-based chatbot initially for people with HIV, to assist pharmacists in considering evidence-based needs. Methods: From December 2022 to December 2023, an online needs-assessment survey evaluated Québec pharmacists’ knowledge, attitudes, involvement, and barriers relative to HIV care, alongside perceptions relevant to the usability of MARVIN-Pharma. Recruitment involved convenience and snowball sampling, targeting National HIV and Hepatitis Mentoring Program affiliates. Results: Forty-one pharmacists (28 community, 13 hospital-based) across 15 Québec municipalities participated. Participants perceived their HIV knowledge as moderate (M = 3.74/6). They held largely favorable attitudes towards providing HIV care (M = 4.02/6). They reported a “little” involvement in the delivery of HIV care services (M = 2.08/5), most often ART adherence counseling, refilling, and monitoring. The most common barriers reported to HIV care delivery were a lack of time, staff resources, clinical tools, and HIV information/training, with pharmacists at least somewhat agreeing that they experienced each (M ≥ 4.00/6). On average, MARVIN-Pharma’s acceptability and compatibility were in the ‘undecided’ range (M = 4.34, M = 4.13/7, respectively), while pharmacists agreed to their self-efficacy to use online health services (M = 5.6/7). Conclusion: MARVIN-Pharma might help address pharmacists’ knowledge gaps and barriers to HIV treatment and care, but pharmacist engagement in the chatbot’s development seems vital for its future uptake and usability.

## 1. Introduction

In Canada, the HIV epidemic is on the rise, with a 24.9% increase in reported cases across the country in 2022, amounting to 1833 new diagnoses [1]. The national rate of new HIV diagnoses was 4.7 per 100,000 people, with Québec slightly exceeding this average at 4.9 per 100,000 [2]. Montréal saw a particularly alarming spike, with a 120% increase in HIV diagnoses between 2021 and 2022, resulting in approximately 310 new cases [3]. This surge is attributed in part to the resumption of HIV testing post-pandemic and the arrival of immigrants from regions where HIV is more prevalent [4].

Managing HIV requires interprofessional efforts, and pharmacists play a significant role in the care of people with HIV (PWH) [5,6,7]. They often serve as first, regular, and trusted points of contact for people seeking medical treatment and advice [8,9,10,11,12], especially in community pharmacies [13]. They support PWH, notably, by providing crucial assistance in the regular refill and management of their antiretroviral therapy (ART).

Generalist pharmacists in Canada can struggle to stay current with HIV treatments and make informed decisions [14]. They can face challenges related to rapidly evolving drug landscapes, constrained time resources, and expanded care roles [15]. These difficulties can be compounded by infrequent interactions with PWH and significantly impair their ability to acquire specialized knowledge and expertise in HIV care, which can, in turn, impact the quality of care they provide.

A range of digital tools are available to support pharmacists in medication management, patient education, and professional communication, including mobile health applications and online clinical references [16,17,18]. These resources provide access to drug information, clinical guidelines, and patient care tips [16,17,18]. Nevertheless, the abundance of online health information complicates the retrieval of specific, reliable data [19], which can make it difficult for pharmacists to provide informed care and navigate treatment options effectively.

Chatbots that leverage artificial intelligence (AI) offer a promising solution to these challenges by facilitating the retrieval of medical information [20,21,22,23]. Chatbots, also known as “conversational agents”, are computer programs designed to mimic human conversation through various platforms, including websites, texting apps, and computer software [22,23,24,25,26,27,28,29,30,31,32,33]. As such, they could provide pharmacists with instant and streamlined access to the most current, validated information on HIV treatments, potentially outperforming web- or app-based tools in these functions. Chatbots could generate personalized advice, aligning with demands from healthcare providers for comprehensive information resources on medication, including, for example, drug–drug interactions and dietary supplements [34,35].

Given pharmacists’ challenges in HIV care delivery, our overarching goal is to adapt an existing AI-based chatbot and connect it with a constantly updated and verified source of information to help pharmacists serve PWH. Since 2020, a team of experts, including physicians, pharmacists, PWH, researchers, and engineers at the McGill University Health Centre (MUHC), developed an innovative AI chatbot called MARVIN for Minimal AntiretRoViral Interference [36]. Running on Facebook Messenger, MARVIN was created as a retrieval-based independent component analysis (ICA) trained to chat with PWH on various aspects of self-management of HIV in both English and French [36]. To configure MARVIN for pharmacists (MARVIN-Pharma), a formal assessment of local pharmacists’ needs is required. With this needs-assessment survey, our objectives are to describe the pharmacists’ knowledge, attitudes, involvement, and barriers in HIV care, as well as their perceptions of MARVIN-Pharma’s usability. Ultimately, this needs assessment aims to inform the adaptation of MARVIN-Pharma to ensure it effectively addresses the identified gaps and challenges in HIV care, enhancing pharmacists’ ability to deliver high-quality care and improving the overall usability and acceptance of the chatbot in clinical practice.

## 2. Methods

### 2.1. Study Design

For this study, we utilized a cross-sectional needs-assessment survey.

#### Conceptual Underpinnings

The Knowledge–Attitudes–Practices (KAP) model underscores the importance of considering the knowledge, attitudes, and practices of a given population, notably, to identify problems, design targeted interventions, and evaluate their effectiveness [37]. Knowledge refers to a person’s understanding of a given topic [38], or their capacity to receive, retain, and utilize information, and may require education [39]. Attitudes reflect a person’s feelings towards a specific topic, and they encompass how individuals react to a given situation [40]. Practice refers to how individuals demonstrate their knowledge and attitudes [38], namely, how they act on these [40]. Based on cognitive, affective, and behavioral theories, the model hypothesizes that relationships exist between these concepts [41]. Specifically, it suggests that knowledge influences attitudes, which in turn influence practices. Further supporting its relevance to our study, the KAP model is commonly used in health research to gather data from patients and practitioners with structured questions in cross-sectional surveys [37,42]. In this study, we assessed all three concepts of this model.

Usability concerns a technology’s ability to be used with effectiveness, efficiency, and satisfaction [43]. The related concepts we measured were perceived acceptability, compatibility, and self-efficacy. Acceptability concerns the degree to which a technology meets its users’ needs, preferences, and expectations; compatibility refers to the extent to which it is consistent with their habits, workflows, and digital environments; and self-efficacy relates to its users’ confidence in their ability to effectively use it. 

**Figure 1:**
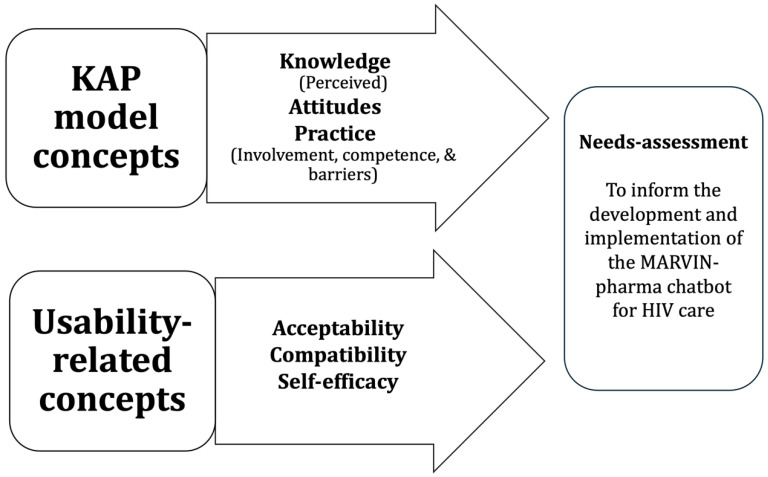
Guiding composite conceptual framework.

A composite framework of the KAP and usability-related concepts guiding this study is shown in Figure 1.

### 2.2. Selection and Enrollment of Participants

#### 2.2.1. Sample Size

Participant recruitment and data collection took place between December 2023 and December 2023. During this time, there were 9385 licensed practicing pharmacists in Québec [44]. Given our desired margin of error (15%), confidence level (95%), and assumed response distribution (50%), we determined our minimum required sample size to be 43 participants. This calculation, performed using an online sample size calculator [45], ensures that our study achieves a minimum precision level of 15 percentage points or less for the half-width of a 95% confidence interval, adequately representing estimated proportions within our population.

#### 2.2.2. Eligibility Criteria

We recruited licensed practicing pharmacists working part- or full-time in the Canadian province of Québec, regardless of their specialized field. We adopted an inclusive approach that acknowledges that all pharmacists may encounter PWH in their practice. 

#### 2.2.3. Recruitment Strategy

We employed a two-fold recruitment approach utilizing convenience [46,47,48] and snowball sampling [48], starting with affiliates of the National HIV and Hepatitis Mentoring Program (Programme National de Mentorat sur le VIH et les Hépatites, or PNMVH). The PNMVH sent out invitations to their mailing list of affiliated pharmacists in Québec that described the study and provided a QR code to the survey as well as the email address of a research team member. The PNMVH also advertised the survey through multiple platforms (PNMVH newsletters, mailing lists, and postings on PNMVH social media accounts). 

Snowball sampling proceeded as follows. Pharmacists who completed the survey were sent an email inviting them to forward the survey link to other pharmacists. The survey also included a prompt for participants to provide the email addresses of other potential respondents [49]. Referred pharmacists were emailed information about the study, including the email address of a study team member.

### 2.3. Data Collection and Analysis

#### 2.3.1. The Survey

##### Sociodemographic and Professional Characteristics

The survey contained items on practice location (city), age, ethnic background, sexual orientation, and gender, as well as years of experience as a pharmacist, type of pharmacy practice setting, and served populations. Additionally, information was collected on Facebook Messenger use and the frequency of health/work application usage on mobile devices. 

##### KAP Model Measures

To measure knowledge, we used a set of questions assessing perceived (subjective) knowledge related to HIV and its pharmaceutical treatment. Perceived knowledge was measured with 5 items on pharmacists’ perceived “preparedness” to advise patients extracted and adapted from a survey that underwent face validity testing with four Arizona pharmacists [50]. Items were answered on a 6-point Likert scale from “Very Unprepared” to “Very Prepared”. Cronback alpha for the 5 items was reported as 0.84.

Attitudes towards PWH and HIV care were measured with 13 attitudinal items also from the previous study’s survey [50]. Respondents recorded their answers on a 6-point Likert scale from “Strongly Disagree” to “Strongly Agree”. 

Practice was assessed with three sets of items. First, 9 questions that measured involvement in HIV care were adapted from a questionnaire that was evaluated for face and content validity, tested for reliability in ten community pharmacies, and used among community pharmacists in Nigeria [51]. These questions were answered with a 5-point Likert scale from “Not Involved” to “Very Much Involved”. Barriers to providing HIV care were measured with 8 items from the same questionnaire. However, we replaced the original multiple-choice response options with a 6-point Likert scale from “Strongly Disagree” to “Strongly Agree”. Competence was evaluated with 4 items from a questionnaire used among Australian pharmacists on dispensing ART [52]. Participants responded with a 6-point Likert scale from “Strongly Disagree” to “Strongly Agree”. 

##### Usability-Related Measures

To limit respondent burden, all three usability-related measures were answered on a 7-point scale of agreement from “Strongly Disagree” to “Strongly Agree”, which differed from the agreement scales of two of the original measures. Acceptability was assessed using the 4-item Acceptability of Intervention Measure (AIM), which has undergone elaborate psychometric testing (Cronbach alpha: 0.85) [53]. Note that the original response scale has 5 points. Compatibility was evaluated with a 4-item subscale of an “Instrument to Measure the Perceptions of Adopting an Information Technology Innovation”, which has demonstrated reliability and evidence of content and construct validity (Cronback alpha: 0.81–0.88) [54]. We slightly modified the original 7-point scale’s labels. To measure self-efficacy to use Internet health services, we used the 4-item Healthcare Technology Self-Efficacy Web subscale [55] with the original response options. It was created with adapted items from existing instruments and submitted to reliability and validity testing (global scale Cronbach alpha: 0.89) [55]. 

### 2.4. Technical Procedures and Survey Administration

We developed the survey in English, which was then translated into French using the forward–backward translation method. A pharmacy student in the research team, who is a native French speaker, performed the initial translation [56]. Subsequently, we retranslated the survey back into English to check for any discrepancies. To ensure linguistic and cultural appropriateness, the translation underwent rigorous pretesting. Two members of the research team, specifically a physician and a pharmacy student, carefully reviewed the French survey for clarity, comprehensibility, and coherence. The pretesting phase aimed to enhance the quality, interpretation, and suitability of the survey. 

Between December 2022 and December 2023, pharmacists completed the survey online through a secure online platform, REDCap© (version 11.1.19 until 15 June 2023, and version 13.1.30 thereafter) [57], which ensured ease of administration and data storage. Participants were offered compensation of 50 CAD to complete the survey. 

### 2.5. Statistical Analysis

We calculated descriptive statistics and reported them in tables. We provide the means and ± standard deviations (SDs) of continuous variables. We summarize categorical variables with frequencies and percentages. Statistical analyses were performed with R statistical software (version 4.2.1) [58]. 

### 2.6. Research Ethics Board (REB)

REB review exemption was obtained from the MUHC REB on 8 September 2022. The project does not qualify as “research” based on the definition outlined in Article 2.1 of the Tri-Council Policy Statement (TCPS2, 2018).

## 3. Results

We recruited 41 pharmacists, and all participants provided complete data.

### 3.1. Sociodemographic and Professional Characteristics

Table 1 presents descriptive sociodemographic information on respondents. Over two-thirds (68%) worked in the Montreal area. The average age of respondents was 36. In terms of ethnic backgrounds, approximately one half (49%) identified as French Canadian while the other half (51%) identified as East Asian, South Asian, Arab, or North African. The gender distribution showed that slightly more women than men were recruited. As to sexual orientation, most (85%) identified as heterosexual.

Professionally, respondents had an average of 10 years of experience. Over two-thirds (68%) worked in community pharmacies, with the remainder working in hospital settings. As to their patient groups, approximately half served women (54%) and LGBTQ+ community members (46%). Over a third provided care to people who use injection drugs (39%) and migrants from HIV-endemic countries (37%). Fewer attended to such groups as the elderly (12%), Indigenous people (12%), and sex workers (10%). Respondents’ patients were approximately 8% PWH, with an additional 12% deemed at high risk of acquiring HIV.

Considering technology usage, on average, respondents used Facebook Messenger regularly for over 10 years, indicating familiarity with the MARVIN chatbot’s current communication platform. Moreover, pharmacists reported, on average, “sometimes” using health or work-related applications on their mobile devices.

### 3.2. Knowledge

#### Perceived Knowledge

Our results indicate a moderate level of perceived knowledge on HIV and its treatment among respondents (Table 2). The average score across the items was 3.74 on 6, almost equivalent to being “somewhat prepared” to advise PWH in the areas evaluated. The highest average item scores (over 4.0) were for preparedness to counsel PWH on HIV transmission and HIV prevention. Meanwhile, the lowest individual item scores (with means between 3.5 and 3.6) concerned adverse drug effects and interactions; HIV treatments; and HIV symptoms and disease progression. These values corresponded to being “somewhat unprepared” to “somewhat prepared” to address these topics. 

### 3.3. Attitudes

For the most part, pharmacists indicated positive attitudes towards PWH and HIV care in the context of their professional work, with a global mean of 4.02 on 6, where a higher mean indicates more favorable attitudes (Table 3). They strongly disagreed with stigmatizing statements (e.g., PWH deserve to have HIV or HIV treatments are not worthwhile) and agreed or strongly agreed with inclusive statements (e.g., I am willing to work with an HIV-positive colleague or providing information about HIV is a professional responsibility). However, pharmacists’ mean attitude toward mandatory continuing education on HIV/AIDS was tepid (M = 3.66; between “Somewhat Disagree” and “Somewhat Agree”).

### 3.4. Practice

#### 3.4.1. Involvement

On average, respondents indicated being “little” involved in HIV care across the services considered (M = 2.08) (Table 4) and were most involved in ART adherence counseling, refilling ART, treatment and monitoring of ART, and referring patients for postexposure prophylaxis (PEP), with mean involvement ratings ranging from 2.3 to 2.8 on 5 (which refer to being “little” to “moderately” involved in these areas). 

#### 3.4.2. Competence

Pharmacists’ global mean score on items related to their perceived competence in HIV care delivery was 3.78/6 (Table 5). On average, they agreed they were sometimes hesitant to dispense ART to PWH and somewhat agreed they were unable to spend enough time counseling PWH. They also somewhat disagreed that they were able to confidently manage the complexities faced by PWH and that they were satisfied with the amount of care they provided them. 

#### 3.4.3. Barriers

Table 6 shows perceived barriers to providing HIV care. Most mentioned barriers were lack of time, of staff resources, of clinical tools, and of information or training on HIV/AIDS services. On average, they at least “somewhat” agreed that they experienced these barriers. 

### 3.5. Usability

Table 7 presents the results on acceptability, compatibility, and self-efficacy. On average, pharmacists were “undecided” about the acceptability of the proposed MARVIN-pharma chatbot (M = 4.34/7) as well as about its compatibility with their work (M = 4.13). In addition, pharmacists showed tempered agreement with their self-efficacy to use Internet health services (M = 5.65).

## 4. Discussion

Drawing on a conceptual framework and existing instruments, this study aimed to better understand the needs of pharmacists in HIV care and their perceptions of the MARVIN-Pharma chatbot (in development) as a tool to reduce their challenges in HIV care. Specifically, it sought to characterize their knowledge, attitudes, and practices tied to HIV care, as well as the chatbot’s usability, in terms of its acceptability, its compatibility, and pharmacist self-efficacy. 

Our findings indicate that pharmacists perceive their HIV knowledge as moderate and face significant barriers, such as lack of time and resources, which hinder their involvement in HIV care. Despite these challenges, there is a generally favorable attitude towards providing HIV care, suggesting a readiness to engage more deeply if provided with the right support tools. However, pharmacists were generally undecided about the acceptability and compatibility of MARVIN-Pharma. Nevertheless, this study highlights the potential of the chatbot to address the identified gaps and barriers, but also the importance of involving pharmacists in its development to ensure its future uptake and usability. 

### 4.1. Knowledge

Our results showed that pharmacists perceived their knowledge to be moderate across the various aspects of HIV care evaluated. As found in past research [50,52], pharmacists felt most prepared to advise patients about HIV transmission and prevention. However, they did not feel fully prepared to advise on HIV treatments [50,52,59]; symptoms and progression of HIV; and adverse drug effects and interactions with ART. Pharmacists’ moderate HIV knowledge, as observed in our study, given their generally limited specialization in this area, reflects the broader trend towards increased HIV knowledge among healthcare professionals in general [50,52,59,60,61,62,63,64,65]. 

Efficiently bridging pharmacists’ knowledge gaps is essential, as their guidance may significantly enhance PWH’s understanding of their treatment [66], potentially boosting ART adherence and lowering HIV transmission rates [67]. MARVIN-Pharma could therefore prioritize content that helps pharmacists provide informed HIV care, such as HIV treatment guidelines and ART regimens, drug interactions, management of medication side effects, and considerations during pregnancy. The chatbot’s connection to a regularly updated ART regimen database like the Canadian HIV/AIDS Pharmacists Network (CHAP) or the Canadian AIDS Treatment Information Exchange (CATIE) seems essential to address the challenge of keeping pace with current information and to streamline access to it. Indeed, Ada Health, Buoy Health, Woebot, and Your.MD are real-world examples of chatbots linked to constantly updated medical databases that demonstrate the feasibility and benefits of such an approach in healthcare [68].

Additionally, MARVIN-Pharma’s eventual ability to deliver just-in-time training and educational content could further enhance pharmacists’ confidence and competence, addressing knowledge gaps as they arise during patient interactions. This practical application ensures the chatbot not only fills existing knowledge gaps but also supports continuous learning, directly linking back to the study’s objective of improving pharmacists’ knowledge in HIV care.

### 4.2. Attitudes

Pharmacists’ attitudes towards PWH and aspects of HIV care were favorable and non-stigmatizing, consistent with previous studies among pharmacists [50,51,62,64]. On average, the participants had been pharmacists for 10 years, which may partially explain these results. Healthcare personnel with greater work experience, especially related to PWH care, are found to have fewer stigmatizing attitudes towards PWH [69].

Our results show that some pharmacists were reluctant towards mandatory continuing education on HIV. This finding increases the relevance of a tool like MARVIN-Pharma for practice [70]. Indeed, AI-driven educational tools have been shown to improve healthcare outcomes by keeping practitioners updated with the latest guidelines and best practices in a user-friendly manner [71,72]. The chatbot, however, is not intended to teach pharmacists the latest guidelines directly. Rather, it will be designed to answer specific questions and provide responses based on these guidelines. Thus, pharmacists will be able to easily retrieve up-to-date information and apply it to real-life situations, enhancing their ability to make informed decisions quickly and effectively during patient care.

This focus on integrating the chatbot seamlessly into pharmacists’ workflows, rather than adding additional educational burdens, directly supports the objective of understanding and addressing pharmacists’ attitudes towards HIV care and ensuring that MARVIN-Pharma aligns with their professional practices.

### 4.3. Involvement

Our findings indicate that pharmacists, overall, were only a “little” involved in HIV care. Their most common activities concerned ART counseling, monitoring, and refilling, as well as PEP referrals. Notwithstanding, pharmacists’ delivery of HIV care remains crucial for extending services beyond specialized settings; it is both feasible and well-received and can help reach underserved populations [73], to enhance antiretroviral adherence, viral suppression, and retention in care [74]. MARVIN-Pharma could assist pharmacists in their important HIV care roles and help compensate for any limited involvement in this area. For example, an adherence counseling module could enable them to perform detailed, interactive assessments of patient adherence, providing personalized advice and interventions based on patients’ responses. Studies have shown that pharmacist-led adherence interventions significantly improve medication adherence and clinical outcomes in HIV patients [75,76,77,78,79]. Additionally, a PEP referral feature could streamline and simplify the referral process, complete with checklists and easily submittable forms to health services. This approach is supported by research indicating that pharmacist-initiated post-exposure prophylaxis (PEP) services increase timely access to PEP and improve adherence to PEP regimens [80,81,82]. 

These practical features demonstrate how MARVIN-Pharma could directly enhance pharmacists’ involvement in HIV care, fulfilling the study’s objective of assessing and improving their engagement in this area.

### 4.4. Competence

Our results indicate that pharmacists experience limited competence in HIV care delivery when managing complex issues faced by patients living with HIV, dispensing ART, and trying to provide sufficient care, underscoring the need for enhanced support and training. 

The competence challenges identified align with those highlighted by past research [52]. Given their findings, some investigators [83] have emphasized the importance of continuous professional development to address knowledge gaps and improve clinical confidence among pharmacists, as well as the need for comprehensive training programs [84] to enhance their ability to manage complex patient needs and improve overall care delivery.

In addition to real-time support for pharmacists’ questions, MARVIN-Pharma could significantly enhance pharmacists’ competence in HIV care by offering tailored interactive learning modules, such as on ART management, drug interactions, and counseling techniques. Connected to a database like CHAP or CATIE, it could incorporate real-time updates on treatment guidelines and emerging data, thus reducing hesitation when dispensing ART. Additionally, it could facilitate just-in-time training, offering educational content and clinical support as pharmacists encounter specific challenges, thereby building confidence and improving the overall quality of HIV care [70].

By addressing these competence challenges through tailored features, MARVIN-Pharma aligns with the study’s ultimate objective of informing the development of tools to enhance pharmacists’ ability to deliver high-quality HIV care, ensuring the practical application of the findings.

### 4.5. Barriers

Mitigating barriers to integrating HIV services into pharmacy practice is crucial for enhancing care. Our study suggests that the pharmacists’ most prominent challenges were limited time, clinical tools, and staff resources, which are known to impact the provision of comprehensive HIV care [51,73,85,86,87]. Importantly, pharmacists also somewhat agreed that they lacked information or training on HIV/AIDS services, which can hinder effective patient counseling and care [14,88,89]. Lesser barriers, including a lack of collaboration with other healthcare professionals, inadequate pharmacy design, patient disinterest in preventive services, and the absence of financial incentives for HIV service provision, have also been reported in prior literature [74,85,90,91].

MARVIN-Pharma could be designed to help address the key barriers identified. By incorporating such features as educational modules [92,93,94,95,96,97], time-management tools [94,96,98,99,100,101], resource access [94,96,100,101,102], collaboration facilitation [99,102,103,104], patient engagement strategies [95,97,100,105], and incentive guidance [95,96,100,101], MARVIN-Pharma could significantly enhance HIV care delivery in pharmacy practice. The literature supports the potential of chatbots to incorporate these features effectively, demonstrating their capacity to overcome various barriers in healthcare. 

Addressing these barriers directly relates to the objective of identifying challenges in HIV care and informing the design of MARVIN-Pharma to overcome them, ensuring the chatbot is practically useful in real-world settings.

### 4.6. Usability

Concerning the acceptability and compatibility of MARVIN-Pharma, pharmacists demonstrated a relatively neutral stance. This may be attributed in part to the hypothetical nature of the chatbot at this stage. Nevertheless, these findings emphasize that, to foster adoption, pharmacists must be engaged in the chatbot’s design to ensure it meets their specific needs and resonates with their professional responsibilities and workflow requirements. Stakeholder involvement and co-design are crucial for the success of mHealth tools. Studies have shown that involving end-users in the design process leads to higher acceptance and more effective implementation [106,107]. By collaborating with pharmacists during the development of MARVIN-Pharma, the tool can be tailored to their circumstances, enhancing its usability and integration into daily practice.

Encouragingly, our study also points to a level of comfort with digital technologies among Quebec pharmacists, aligned with broader research showing openness to digital tools among pharmacists [16,17,18,108,109,110,111,112,113,114]. The sample’s self-efficacy with using Internet health services, their average 10 years of using Facebook Messenger, and their occasional use of health or work-related applications on their mobile devices indicate a potential facility to take up a tool like MARVIN-Pharma.

Emphasizing the importance of user involvement and the need for further development based on pharmacists’ feedback highlights how the findings align with the objective of assessing MARVIN-Pharma’s usability to ensure its successful implementation in practice.

## 5. Limitations of This Study

This study has several limitations. Despite employing multiple recruitment strategies, its sample size was small (*n* = 41 pharmacists). We were thus unable to examine the effects of potential confounders (e.g., age, gender, and professional experience level). While snowball sampling may have helped reach a broader range of participants, self-selection and sampling bias may still affect the generalizability of the obtained results. For instance, the predominance of pharmacists from Montreal may not accurately represent the broader population of pharmacists in the province, and the sample’s average of 10 years of pharmacy experience and slight overrepresentation of females suggest a relatively experienced and gender-skewed group. We did, however, largely attain our sample size target to help ensure adequate statistical precision. 

While this study relied on participant self-report, potentially introducing risks of response, recall, social desirability, and confirmation bias, the consistency of results obtained with respect to previous literature is encouraging in this regard.

This study’s quantitative design and limited scope mean the nuances and depth of pharmacists’ experiences and needs were not explored. A more comprehensive perspective will be achieved with the analysis of qualitative interview data collected as part of the larger mixed methods research project in which this study is embedded.

## 6. Conclusions

Our needs-assessment survey of Québec pharmacists indicates that while they have moderate HIV-related knowledge and positive attitudes towards providing HIV services in pharmacy practice, their involvement in HIV care remains limited. Despite pharmacists’ apparent capacity to adopt digital tools like MARVIN-Pharma, knowledge gaps and barriers in HIV treatment and management were identified. Although MARVIN-Pharma has the potential to address these challenges, its success will likely depend, in part, on achieving adequate perceived acceptability and compatibility with pharmacists’ work, on which they were undecided in this study. The findings suggest that buy-in for MARVIN-Pharma is not assured and that it must be developed with continuous pharmacist feedback and responsiveness to their specific needs.

## Figures and Tables

**Table 1:** Participating pharmacists’ sociodemographic and professional characteristics (*n* = 41).

Characteristic	*n* (%)	Mean ± SD (Range)
City of work		
Montreal	28 (68%)	
Other cities	13 (32%)	
Age	-	36 yrs. ± 8 (23–52)
Ethnicity		
French Canadian	20 (49%)	
Other	21 (51%)	
Sexual orientation		
Heterosexual	35 (85%)	
Homosexual	3 (7%)	
Bisexual	3 (7%)	
Gender		
Female	25 (61%)	
Male	16 (39%)	
Years of pharmacy experience	-	10 yrs. ± 8 (1–30)
Main setting of pharmacy practice		
Community	28 (68%)	
Hospital	13 (32%)	
Patient groups seen by pharmacists		
Women	22 (54%)	
People from the LGBTQ+ community	19 (46%)	
People who use injection drugs	16 (39%)	
People from countries where HIV is endemic	15 (37%)	
HIV-negative partners of PWH	8 (20%)	
Indigenous people	5 (12%)	
Elderly	5 (12%)	
Sex workers	4 (10%)	
Incarcerated people	1 (2%)	
Dialysis patients with frequent transfusions	1 (2%)	
Percentage of patients with HIV	-	8 ± 15% (0–75%)
Percentage of patients at high risk of HIV	-	12 ± 15% (0–66%)
Years of using Facebook Messenger regularly	-	11 yrs. ± 3 (1–20)
Frequency of using applications related to health/work on pharmacists’ mobile devices	-	2 ± 1 (0–4) *

* Frequency of use was rated on a five-point scale from 0 = “Never” to 4 = “Very Frequently”.

**Table 2:** Pharmacists’ perceived knowledge to counsel people with HIV in various areas of HIV care.

Area	Response * *n*						Mean ± SD
	1	2	3	4	5	6	
HIV prevention	1	3	10	11	12	4	4.02 ± 1.20
HIV transmission	1	3	8	14	11	4	4.05 ± 1.17
HIV/AIDS symptoms and disease progression	1	6	16	12	1	5	3.51 ± 1.21
HIV treatments	1	4	20	10	1	5	3.51 ± 1.17
Adverse effects and drug–drug interactions with HIV treatments	1	4	19	8	4	5	3.61 ± 1.23
Global average							3.74 ± 1.20

* 1 = Very Unprepared; 2 = Unprepared; 3 = Somewhat Unprepared; 4 = Somewhat Prepared; 5 = Prepared; 6 = Very Prepared.

**Table 3:** Pharmacists’ attitudes toward people with HIV and their related care.

Statement	Response * *n*						Mean ± SD
	1	2	3	4	5	6	
Providing information about HIV/AIDS is part of a pharmacist’s professional responsibility	0	0	2	7	14	18	5.17 ± 0.88
I have time while at work to answer questions about HIV/AIDS	2	8	8	9	7	7	3.78 ± 1.49
People who are educated about how HIV is transmitted sexually will change their risky behavior	1	1	6	19	11	3	4.15 ± 1.00
People who are educated about how HIV is transmitted by contaminated needles will change their risky behavior	2	2	9	19	5	4	3.85 ± 1.16
Some people who have HIV/AIDS deserve to have the disease **	34	5	2	0	0	0	5.78 ± 0.52
Drugs to treat HIV/AIDS are too expensive for the pharmacy to keep in stock	1	6	16	8	6	4	3.59 ± 1.25
Approved treatments for HIV/AIDS simply delay the inevitable and may not be worthwhile **	29	9	2	1	0	0	5.61 ± 0.69
I would provide information about a patient’s HIV status to healthcare providers other than the prescribing physician	22	5	7	5	1	1	2.05 ± 1.34
I might become infected with HIV from contact with an HIV/AIDS patient when I am working **	17	10	8	2	3	1	2.90 ± 1.14
Many of my patients are at high risk for HIV/AIDS	14	16	5	3	3	0	2.15 ± 1.18
I might be sued if I unknowingly advise an HIV/AIDS patient with incomplete medication information	1	7	8	6	12	7	4.02 ± 1.44
I am willing to work with a coworker who has HIV/AIDS	0	0	0	4	9	28	5.59 ± 0.66
Continuing education on HIV/AIDS should be mandatory	3	4	11	11	10	2	3.66 ± 1.28
Global mean							4.02 ± 1.64

* 1 = Strongly Disagree; 2 = Disagree; 3 = Somewhat Disagree; 4 = Somewhat Agree; 5 = Agree; 6 = Strongly Agree. ** The reversal process for this item converts the original score x to the reversed score 7 − x to calculate the global mean.

**Table 4:** Pharmacists’ involvement in HIV care services.

HIV Care Service	Response * *n*					Mean ± SD
	1	2	3	4	5	
Pharmaceutical care to HIV patients	9	23	4	1	4	2.22 ± 1.12
Adherence counseling for HIV/AIDS patients	9	15	9	3	5	2.51 ± 1.25
Referral of patients for postexposure prophylaxis (PEP)	10	15	12	3	1	2.27 ± 0.99
Refill of ART	15	9	7	8	2	2.34 ± 1.28
Stocking of ART	19	7	10	4	1	2.05 ± 1.15
Social responsibility to HIV patients	18	12	6	3	2	2.00 ± 1.15
Treatment and monitoring of ART	13	12	10	2	4	2.32 ± 1.24
Provision of a PEP test for a patient	31	8	1	0	1	1.34 ± 0.75
Home delivery of ART	24	10	3	3	1	1.71 ± 1.04
Global mean						2.08 ± 1.12

* 1 = Not Involved; 2 = Little Involvement; 3 = Moderately Involved; 4 = Very Involved; 5 = Very Much Involved. PEP = post-exposure prophylaxis; ART = antiretroviral therapy.

**Table 5:** Pharmacists’ perceived competence in the delivery of HIV care.

Statement	Response * *n*						Mean ± SD
	1	2	3	4	5	6	
I believe I am able to confidently manage the complex issues and experiences faced by patients living with HIV	10	10	11	4	2	4	2.76 ± 1.53
I feel I am unable to spend sufficient time counseling patients living with HIV **	5	7	11	14	1	3	3.81 ± 1.31
I am satisfied with the amount of care I provide to patients living with HIV	4	12	11	8	2	4	3.10 ± 1.39
I sometimes feel hesitant to dispense HIV medicines to patients living with HIV **	27	8	3	3	0	0	5.44 ± 0.91
Global mean							3.78 ± 1.31

* 1 = Strongly Disagree; 2 = Disagree; 3 = Somewhat Disagree; 4 = Somewhat Agree; 5 = Agree; 6 = Strongly Agree. ** The reversal process for this item converts the original score x to the reversed score 7 − x to calculate the global mean.

**Table 6:** Pharmacists’ perceived barriers to providing HIV care.

Barrier	Response * *n*						Mean ± SD
	1	2	3	4	5	6	
Lack of clinical tools (i.e., HIV test kits)	0	3	6	16	11	5	4.22 ± 1.07
Lack of collaboration with other healthcare professionals	3	7	9	14	5	3	3.49 ± 1.31
Lack of information or training on HIV/AIDS services	0	6	5	19	5	6	4.00 ± 1.19
Lack of staff resources	1	4	1	14	16	5	4.34 ± 1.18
Absence of financial compensation	2	9	8	8	8	6	3.71 ± 1.49
Lack of space/inadequate physical design of the pharmacy	5	6	6	9	10	5	3.68 ± 1.57
Lack of time	3	1	1	9	14	13	4.68 ± 1.39
Patients are not interested in preventive services	3	9	12	13	3	1	3.17 ± 1.15

* 1 = Strongly Disagree; 2 = Disagree; 3 = Somewhat Disagree; 4 = Somewhat Agree; 5 = Agree; 6 = Strongly Agree.

**Table 7:** Pharmacists’ perceived acceptability and compatibility with the chatbot and self-efficacy to use Internet health services.

	Response * *n*							Mean ± SD
	1	2	3	4	5	6	7	
Acceptability								
MARVIN Pharma Chatbot meets my approval	1	1	1	31	3	3	1	4.15 ± 0.95
MARVIN Pharma Chatbot is appealing to me	1	0	1	26	8	4	1	4.37 ± 0.92
I like MARVIN Pharma Chatbot	1	0	1	30	7	1	1	4.20 ± 0.80
I welcome MARVIN Pharma Chatbot	1	0	0	23	7	8	2	4.63 ± 1.10
Global mean								4.34 ± 0.95
Compatibility								
Using MARVIN Pharma Chatbot is compatible with all aspects of my work	1	0	2	27	7	3	1	4.27 ± 0.94
Using MARVIN Pharma Chatbot is completely compatible with my current situation	1	0	5	28	3	4	0	4.07 ± 0.90
I think that using the MARVIN Pharma Chatbot fits well with the way I like to work	1	0	7	26	3	3	1	4.07 ± 0.90
Using MARVIN Pharma Chatbot fits into my work style	1	1	3	27	6	2	1	4.12 ± 0.97
Global mean								4.13 ± 0.95
Self-efficacy								
It is easy for me to use internet health services	0	1	0	0	15	16	9	5.76 ± 0.96
I feel uncomfortable using internet health services **	11	16	4	0	4	5	1	5.27 ± 1.81
I am very confident in my abilities to use internet health services	0	1	0	0	15	14	11	5.81 ± 0.99
I would be able to use internet health services without much effort	0	0	1	2	14	13	11	5.76 ± 0.98
Global mean								5.65 ± 1.24

* 1 = Strongly Disagree; 2 = Disagree; 3 = Somewhat Disagree; 4 = Undecided; 5 = Somewhat Agree; 6 = Agree; 7 = Strongly Agree. ** The reversal process for this item converts the original score x to the reversed score 8 − x to calculate the global mean.

## Data Availability

The data sets generated and analyzed for this study are available from the corresponding author (B.L.) on reasonable request.

## Abbreviations

AI	Artificial intelligence
ART	Antiretroviral therapy
ICA	Independent component analysis
KAP	Knowledge–Attitudes–Practices
MARVIN	Minimal AntiretRoViral Interference
MARVIN-Pharma	Minimal AntiretRoViral Interference for Pharmacists
MUHC	McGill University Health Centre
N	Number
PNMVH	Programme National de Mentorat sur le VIH et les Hépatites (National HIV and Hepatitis Mentoring Program)
PWH	People with HIV
REB	Research Ethics Board
SD	Standard deviation
TCPS	Tri-Council Policy Statement
